# The Global Burden of Disease project: an online tool to compare disease impact across geographical regions over time

**DOI:** 10.36416/1806-3756/e20240399

**Published:** 2024-12-20

**Authors:** Rogelio Perez-Padilla, Cecilia Maria Patino, Juliana Carvalho Ferreira

**Affiliations:** 1. Methods in Epidemiologic, Clinical, and Operations Research-MECOR-program, American Thoracic Society/Asociación Latinoamericana del Tórax, Montevideo, Uruguay.; 2. Instituto Nacional de Enfermedades Respiratorias, Ciudad de Mexico, Mexico.; 3. Department of Population and Public Health Science, Keck School of Medicine, University of Southern California, Los Angeles (CA) USA.; 4. Divisão de Pneumologia, Instituto do Coração, Hospital das Clínicas, Faculdade de Medicina, Universidade de São Paulo, São Paulo (SP) Brasil.

## PRACTICAL SCENARIO

Investigators from the *Instituto Nacional de Enfermedades Respiratorias* (INER, National Institutes of Respiratory Diseases) in Mexico aimed to analyze the burden of respiratory diseases in the adult Mexican population over a 30-year period. They needed trustworthy information about the incidence and mortality of several diseases over a large period and opted to use data from The Global Burden of Diseases (GBD) initiative. They found that respiratory diseases are responsible for approximately 30% of deaths among adults in Mexico, and that mortality due to chronic respiratory diseases has been rising since 1990 across both genders.^1^

## THE GBD INITIATIVE

The GBD initiative, launched in 1991 by the World Bank and the World Health Organization, is a comprehensive framework that generates standardized health information worldwide.^2^ It is used to identify health disparities and priorities, inform evidence-based public health decisions, and measure their impact. Managed by the Institute for Health Metrics and Evaluation (IHME) at the University of Washington in the United States, the GBD’s leading articles have been published in Lancet.^3^

The GBD’s data allows comparisons across different regions, countries, populations, genders, and age groups from 1990 to 2021. Before the GBD, no consistent global reporting of the prevalence and mortality of diseases existed, thus several countries, especially low- and middle-income countries, lacked accurate health statistics.

The GBD project has been instrumental in promoting innovation in reporting disease impact. It introduced the concept of disability-adjusted life years (DALYs), a comprehensive measure of health that combines years of life lost by premature death (YLL) and years of life lived with disability (YLD). This approach, which considers both quantity (mortality) and quality (morbidity) of life, has significantly expanded our understanding of disease burden. 

The GDB is easily accessible to clinicians, clinical researchers, epidemiologists, public health professionals, politicians, and the general public. It can be consulted online and includes tables, graphs, and open-access downloadable data. However, the GBD does not provide estimates for all diseases or risk factors, only for the most relevant ones, although the number has been increasing. Chart 1 shows the main characteristics of the GBD.

**Chart 1:** Main characteristics of Global Burden of Disease initiative of interest for respiratory diseases.

Scope of GBD
• Data on 371 diseases and injuries, 288 causes of death, and 88 risk factors; such data can be stratified by age and sex across 204 countries and territories from 1990 to 2021
• Vital statistics (births and deaths), household surveys (from health surveys, hospital discharge data, disease registers, annual health reports (countries and WHO)
Main GBD estimates
• Deaths (and causes, all-cause mortality), years of life lost (YLL), prevalence, incidence, population, and causes of injuries and impairment. Estimates by number, crude rate and age-adjusted rates, percentage, probability of death across countries, world regions (WHO, World Bank income levels), and over time (1990-2021)
• Life expectancy (LE), life tables (LTs), healthy life expectancy (HALE), years lived with disability (YLD), disability-adjusted life year (DALYs)
Sources and methodology for the generation of estimates
• Input from expert groups, World Health Organization, World Bank, UNICEF, and expert collaborators from a large variety of countries
• Bayesian statistical models adjusted for underreporting and poor data quality
Data retrieval tools
• The Global Health Data Exchange (GHDx) yields tables by cause, risk factor, sex, age, and year (1990-2021), as well as graphs and downloadable results. (https://vizhub.healthdata.org/gbd-results/
• The GBD 2021 Socio-Demographic Index 1950-2021 provides a composite indicator of development status strongly correlated with health outcomes. (https://ghdx.healthdata.org/record/global-burden-disease-study-2021-gbd-2021-socio-demographic-index-sdi-1950%E2%80%932021)
• The GBD 2021 Causes of Death and Nonfatal Causes Mapped to ICD Codes provides tables that contain ICD codes, for both ICD-9 and ICD-10, mapped to GBD 2021 causes of death and nonfatal causes. • https://ghdx.healthdata.org/record/ihme-data/gbd-2021-cause-icd-code-mappings

GBD: Global Burden of Disease; and ICD: International Classification of Diseases.

The GBD project covers a range of respiratory diseases and their risk factors.^1^ These include chronic respiratory diseases, such as COPD, interstitial lung diseases, sarcoidosis, pneumoconiosis, and asthma. It includes respiratory infections, such as COVID-19, such as lower and upper respiratory infections, and tuberculosis, and respiratory neoplasms, such as lung and head-and-neck cancers. Commonly reported risk factors for respiratory diseases include air pollution (outdoor particulate matter and ozone, household air pollution, occupational exposures), active and passive tobacco smoking, and infections.

The GBD has been regularly updated since 1990, and most data are available at the Global Health Data Exchange website without cost (GHDx, https://ghdx.healthdata.org). It is a clinician-friendly source of information, including tables, graphs, and illustrations on the status of disease burden, which are key for presentations and introductions to thesis or manuscripts.

## KEY MESSAGES

- The GBD is an open-access reliable database used to describe and compare estimates of respiratory health indicators across countries and regions over time.
- It is helpful for clinicians, epidemiologists, and public health officers.
- Empiric validation of the estimates should be sought, especially for areas lacking sufficient health information.
